# Childhood Atopic Diseases and Early Life Circumstances: An Ecological Study in Cuba

**DOI:** 10.1371/journal.pone.0039892

**Published:** 2012-06-29

**Authors:** Suzanne D. van der Werff, Katja Polman, Maiza Campos Ponce, Jos W. R. Twisk, Raquel Junco Díaz, Mariano Bonet Gorbea, Patrick Van der Stuyft

**Affiliations:** 1 Department of Health Sciences, VU University Amsterdam, Amsterdam, The Netherlands; 2 Department of Biomedical Sciences, Prince Leopold Institute of Tropical Medicine, Antwerp, Belgium; 3 Department of Epidemiology and Biostatistics, EMGO Institute of Health and Care Research, VU University Medical Center, Amsterdam, The Netherlands; 4 National Institute of Hygiene, Epidemiology and Microbiology, Havana, Cuba; 5 Department of Public Health, Prince Leopold Institute of Tropical Medicine, Antwerp, Belgium; 6 Department of Public Health, Ghent University, Ghent, Belgium; Erasmus Medical Center, The Netherlands

## Abstract

**Background:**

Children are especially vulnerable during periods of resource shortage such as economic embargoes. They are likely to suffer most from poor nutrition, infectious diseases, and other ensuing short-term threats. Moreover, early life circumstances can have important consequences for long-term health. We examined the relationship between early childhood exposure to the Cuban economic situation in the nineties and the occurrence of atopic diseases later in childhood.

**Methodology/Principal Findings:**

A cross-sectional study of 1321 primary schoolchildren aged 4–14 was conducted in two Cuban municipalities. Asthma, allergic rhinoconjunctivitis and atopic dermatitis were diagnosed using the International Study of Asthma and Allergies in Childhood questionnaire. Children were divided into three groups of exposure to the economic situation in the nineties according to birth date: (1) unexposed; (2) exposed during infancy; (3) exposed during infancy and early childhood. Associations were assessed using multiple logistic regression models. Exposure during infancy had a significant inverse association with the occurrence of asthma (OR 0.56, 95%CI 0.33–0.94) and allergic rhinoconjunctivitis (OR 0.46, 95%CI 0.25–0.85). The associations were stronger after longer exposure, i.e. during infancy and early childhood, for asthma (OR 0.40, 95% CI 0.17–0.95) and allergic rhinoconjunctivitis (OR 0.29, 95%CI 0.11–0.77). No significant associations were found for atopic dermatitis.

**Conclusions/Significance:**

Exposure to the economic situation in the nineties during infancy and early childhood was inversely associated with asthma and allergic rhinoconjunctivitis occurrence later in childhood. We hypothesize that factors related to this period, such as infectious diseases and undernutrition, may have an attenuating effect on atopic disease development. The exact cause and underlying mechanisms need to be further elucidated.

## Introduction

Economic crises can have a negative impact on health [1], [2]. In Cuba a period of great economic difficulty, known as the “Special Period”, affected the country in the early 1990 s. The United States instated an economic blockade against the island in 1961. This embargo, along with the collapse of the Soviet Union and the Eastern European socialist block in the late 1980 s, reduced Cuban foreign trade by 80%. Multiple economic constraints evolved which further deteriorated when the U.S. intensified their sanctions in the early 1990 s. The situation improved during 1995–1996 and especially from 1996 on, but complete economic recovery was not reached until after 2000 [3]–[6].

The economic problems affected the health of the Cuban population in various ways, due to a sudden shortage of essential products such as food, energy, drugs and medical equipment [3]–[5]. Approximately half of the food needed to meet caloric and protein needs was imported. Therefore, the decline in food imports combined with an already low food production within Cuba resulted in a 40% reduction in the availability of nutritional energy per capita [3]–[6]. Furthermore, diet quality, composition and patterns were affected [5]. Vitamin deficiencies led to an increase of anaemia in pregnant women and infants and an epidemic of optic neuropathy predominantly among males [3], [7], [8]. The incidence of tuberculosis increased as did the mortality rates from infectious and parasitic disorders, and influenza and pneumonia [3]. Although the economic crisis in Cuba was severe, the harmful effects on general public health were reduced to a minimum due to appropriate economic and social measures taken by the government to counter the crisis [9]. For example, vulnerable groups like children, women, and the elderly were prioritized for protection against nutritional deficiencies [3].

Children under the age of five are especially vulnerable during economic embargoes. They are likely to suffer most from poor nutrition, increased infectious disease risk, and other ensuing short-term threats [2], [10]. Moreover, early life circumstances may have serious consequences for long-term health, such as cardiovascular and other chronic diseases [11], [12]. Previous research on the health impact of economic crises mostly focused on morbidity, mortality, impact on health care, and nutritional status during and shortly after a crisis [13]–[16]. Longer term consequences, like altered chronic disease occurrence, have been studied less and mostly in adults [11].

In this ecological study, we examined the relationship between early childhood exposure to the Cuban economic situation in the nineties and the occurrence of atopic diseases later in childhood. Possible contributing factors and underlying mechanisms for this association will be discussed.

## Methods

### Study Design

A cross-sectional study was conducted in 1321 primary schoolchildren of two municipalities in Cuba: San Juan y Martínez (SJM) in Pinar del Rio (December 2003), a province in the west of Cuba, and Fomento in Sancti Spiritus (May 2004), a province in the centre of Cuba. Rural and urban primary schools were randomly selected from SJM (N = 5) and Fomento (N = 14). All children were included in the study, i.e. 398 children from SJM and 923 children from Fomento. Data on atopic diseases, other relevant health and environmental factors, and demographic characteristics were collected. Further details have been described elsewhere [17], [18].

### Ethics Statement

Informed written consent was obtained from the parents or guardians of each participating child. This study is part of a larger investigation on atopic diseases and helminth infections in Cuban children, for which approval was obtained from the Ethical Committees of the Prince Leopold Institute of Tropical Medicine in Antwerp, Belgium, the Pedro Kourí Institute (IPK) of Tropical Medicine and the National Institute for Hygiene, Epidemiology and Microbiology (INHEM) in Havana, Cuba.

### Atopic Diseases

Atopic disease occurrence, i.e. asthma, allergic rhinoconjunctivitis and atopic dermatitis, was determined by means of the standard Spanish version of the International Study of Asthma and Allergies in Childhood (ISAAC) questionnaire [19], whereby a parent or guardian of each child was interviewed by a trained local team member. ISAAC definitions of atopic diseases were used in this study: current asthma, shortened to ‘asthma’ throughout the text, was defined as an affirmative answer to the second ISAAC core asthma question on current wheeze [20]; allergic rhinoconjunctivitis was defined as an affirmative answer to the second and third core questions of the ISAAC modules on rhinitis [21]; and atopic dermatitis was defined as an affirmative answer to the second and third core questions of the ISAAC modules on eczema [22].

### Exposure to Economic Situation

Exposure to the Cuban economic situation in the nineties was determined using the child’s date of birth. None of the children in our study group were born before 1990. The gross domestic product (GDP) dropped dramatically starting in 1990 [4]. As the situation improved during 1995–1996 [5], [6], the exposure period was set from January 1^st^ 1990 until January 1^st^, 1996. Children were divided into three groups: (1) exposed during infancy (<24 months) *and* early childhood (2–6 year), i.e. born before January 1^st^, 1994; (2) exposed *only* during infancy, i.e. born from January 1^st^, 1994 till January 1^st^, 1996; and (3) unexposed, i.e. born from January 1^st^, 1996 and later.

### Covariates

Demographic variables considered were sex, age (in years), municipality (SJM vs. Fomento) and area of residence (rural vs. urban). Socio-economic variables were monthly household income (250 pesos (≈ 7 euro)/month or less vs. more than 250 pesos/month) and education level of the parents (less than grade 12 vs. grade 12 or higher). Perinatal variables considered were low birth weight (LBW), i.e. a birth weight less than 2500 gram (yes or no), and premature birth, i.e. a gestational age of less than 37 weeks (yes or no). The variables included regarding the first year of life were breastfeeding for more than six months (yes or no) and antibiotics use (yes or no). All these variables were collected by means of a structured parental questionnaire.

### Statistical Analysis

Statistical analyses were conducted using SPSS (SPSS Inc., Chicago, IL, USA) version 17.0 for Windows. A *P*-value of 0.05 or less was regarded as statistically significant.

Characteristics of the study population are given as numbers and percentages, except age which is given as median and its interquartile range (IQR). Differences in the covariates between the three exposure groups were tested using the Chi-square test, except age for which the Kruskal-Wallis test was used. Univariate logistic regression models were performed to assess crude associations between exposure to the economic situation in the nineties and atopic disease outcomes with the unexposed group as the reference. Subsequently, the covariates were entered using a stepwise forward approach to examine possible confounding or effect modification. Only relevant confounders, i.e. which satisfied a change-in-estimate criterion of ≥10% [23], and significant effect modifiers were included in the final multiple logistic regression models.

To test the robustness of the results, sensitivity analyses were performed: the two cut-off dates that distinguished the three groups were shifted forward for three and six months, and the ‘transition groups’ with a range of six months before and after both cut-off dates were removed, and associations were re-assessed.

## Results

All 1321 children were included in the analysis. The participating children were aged 4 till 14 (median 8 years) and consisted of 678 boys (51%) and 643 girls (49%). The response rate to the questionnaires was 100%. Characteristics of the study population according to their exposure to the economic situation in the nineties are shown in Table 1. The three groups significantly differed from each other on age, municipality, and education level of the mother.

**Table 1:** Characteristics of study population according to exposure to the Cuban economic situation in the nineties.

	Unexposed		Exposed during infancy		Exposed during infancy and early childhood		N	*P*-value*
Number of children	541		400		380		1321	
Sex (male)	265	(49.0%)	202	(50.5%)	211	(55.5%)	1321	0.14
Age (years)	6	(1)	9	(1)	11	(1)	1321	**<0.001**
Municipality (Fomento)	375	(69.3%)	259	(64.8%)	289	(76.1%)	1321	**0.003**
Area of residence (urban)	280	(51.8%)	210	(52.5%)	200	(52.6%)	1321	0.96
Family income (>250 peso/month)	249	(46.2%)	182	(45.8%)	162	(43.1%)	1312	0.62
Education level father (≥12 grades)	239	(44.8%)	181	(46.5%)	159	(42.4%)	1297	0.51
Education level mother (≥12 grades)	261	(48.6%)	204	(51.1%)	151	(39.9%)	1314	**0.004**
LBW (<2500 g)	51	(9.5%)	51	(13.0%)	31	(8.3%)	1304	*0.07*
Premature birth (<37 weeks)	37	(6.8%)	29	(7.3%)	23	(6.1%)	1321	0.80
Breastfeeding (>6 months)	284	(52.7%)	198	(49.7%)	181	(47.9%)	1315	0.34

Data are given as numbers and percentage, except age which is given as median (IQR).

Statistically significant differences are given in bold and borderline significant differences in italic.

* Chi-square test for difference between the three exposure groups, expect for age which was done by Kruskal-Wallis test.

**Figure 1:**
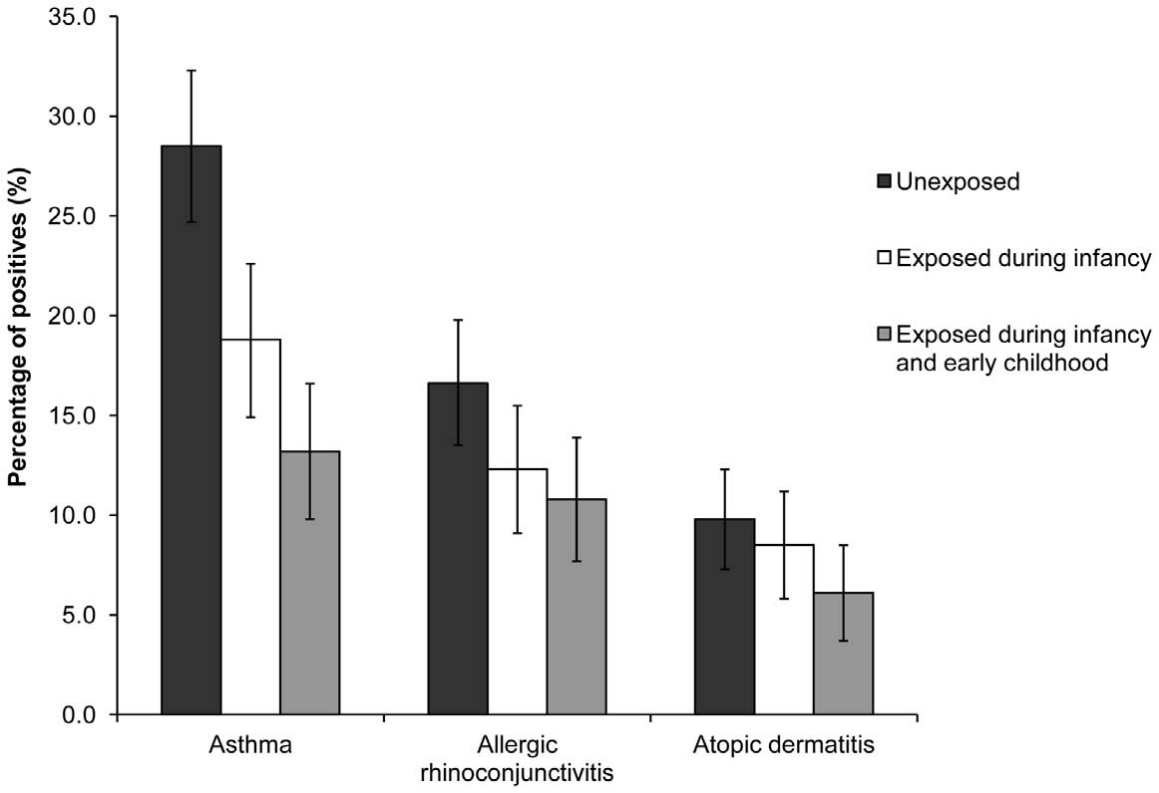
Atopic diseases according to exposure status to the Cuban economic situation in the nineties. (asthma resp. 28.5%, 18.8% and 13.2%; allergic rhinoconjunctivitis resp. 16.6%, 12.3% and 10.8%; atopic dermatitis resp. 9.8%, 8.5% and 6.1%).

Based on the parental ISAAC questionnaire 279 of the 1321 children (21.1%) were diagnosed with asthma, 180 (13.6%) with allergic rhinoconjunctivitis, and 110 (8.3%) with atopic dermatitis. The percentages of positives for the different atopic diseases according to exposure status (Figure 1) show that (longer) exposure to the economic situation in the nineties corresponds with decreased atopic disease occurrence.

Table 2 shows the crude and adjusted ORs of the two groups exposed to the economic situation in the nineties for the different atopic diseases compared to the unexposed group. The adjusted ORs confirmed the observations of Figure 1, with significant associations between exposure and asthma and allergic rhinoconjunctivitis. These associations tended to be stronger for longer exposure, i.e. during both infancy and early childhood. However, the differences between the two exposure groups were not significant. No significant association was found between exposure and atopic dermatitis.

**Table 2:** Crude and adjusted odds ratio’s (OR) with 95% confidence intervals (CI) of exposure to the Cuban economic situation in the nineties for the different atopic diseases.

		Crude OR (95% CI)	*P*-value	Adjusted OR (95% CI)*	*P*-value
Asthma					
	*Unexposed*	1.0		1.0	
	*Exposed during infancy*	0.58 (0.42–0.79)	**0.001**	0.56 (0.33–0.94)	**0.03**
	*Exposed during infancy and early childhood*	0.38 (0.27–0.54)	**<0.001**	0.40 (0.17–0.95)	**0.04**
Allergic rhinoconjunctivitis					
	*Unexposed*	1.0		1.0	
	*Exposed during infancy*	0.70 (0.48–1.02)	*0.06*	0.46 (0.25–0.85)	**0.01**
	*Exposed during infancy and early childhood*	0.61 (0.41–0.90)	**0.01**	0.29 (0.11–0.77)	**0.01**
Atopic dermatitis					
	*Unexposed*	1.0		1.0	
	*Exposed during infancy*	0.86 (0.55–1.35)	0.51	1.58 (0.72–3.44)	0.25
	*Exposed during infancy and early childhood*	0.59 (0.36–0.99)	**0.04**	1.86 (0.52–6.65)	0.34

Statistically significant associations are given in bold and borderline significant associations in italic.

* Adjusted for age & municipality.

The sensitivity analysis by shifting forward the cut-off dates confirmed the general trend found for asthma and allergic rhinoconjunctivitis (Table S1). As expected, removing the transition groups slightly strengthened most associations (Table S2). Only for atopic dermatitis the results were altered after sensitivity analysis, confirming the instability of the original results for atopic dermatitis due to the small group sizes (Table S1 and S2).

## Discussion

So far most studies on the impact of economic crises on health have focussed on immediate health consequences during or shortly after the crisis [4], [5], [13]–[16]. Longer term health consequences like chronic diseases have been investigated to a lesser extent. Here, we studied the effects of the Cuban economic situation in the nineties on the occurrence of atopic diseases in Cuban children 10 years later. We observed that exposure to the economic circumstances during infancy and early childhood had an attenuating effect on atopic disease development later in childhood.

A few limitations of this study should be noted. Firstly, all ecological studies are potentially prone to the so-called ‘ecological fallacy’, and our study findings should thus be interpreted cautiously. Although we checked for potential confounders, we cannot exclude that unknown or unmeasured contemporary factors not related to the economic situation may have influenced the study results. Our atopic disease data are based on the ISAAC questionnaire, which has become the standard diagnostic method in childhood epidemiology of atopic diseases worldwide [19]. Nevertheless, questionnaires have important inherent limitations, such as information and recall bias, which should be kept in mind when interpreting the data. Also, an independent trend of increasing atopic diseases prevalence over time, like in the Western world, cannot be ruled out [24]. Moreover, we used GDP to define the period of exposure, as this seems to be the most objective and well-documented proxy for exposure to the Cuban economic situation in the nineties. However, we do realize that GDP is an indirect measure of exposure. Thus, the possibility remains that our conclusions are based on an inadequate assumption of how GDP translates into exposure to the economic circumstances. Also, we did not take severity of the economic situation into account, e.g. the first years of the exposure period may have been less severe than the following years, resulting in differences in impacts on health in infancy and childhood. However, such data are scarce, and usually report on one aspect, such as per capita calorie consumption or low birth weight prevalence [3], [5], so we could not to take this aspect into account. Although we corrected for age, we are aware that this cannot completely adjust for age-related trends in the prevalence of atopic diseases. However, the adjusted effects we found show that older children are less likely to have asthma and allergic rhinoconjunctivitis and more likely to have atopic dermatitis. Therefore, these effects are different or even opposite of the normal age trends, suggesting that the effect of the economic circumstances is genuine. Finally, since it is difficult to determine an exact end point for the exposure period, the chosen cut-off date (January 1^st^, 1996) is somewhat arbitrary and therefore possible misclassification cannot be ruled out. Nevertheless, we believe that our results are robust, as demonstrated by the sensitivity analyses, and do indicate an inverse association between atopic diseases in today’s Cuban schoolchildren and exposure to the economic circumstances in the nineties.

The associations found suggest an attenuating effect of factors related to the economic situation on atopic disease development. Below we speculate on potential factors and mechanisms underlying the observed associations.

During Cuba’s Special Period there were two important health trends. One was a small and temporary rising in mortality rates from infectious and parasitic disorders and increased incidence of tuberculosis [3], [25]. Even though we do not have exact data on other infectious disease incidences, it is very likely that these were elevated as well, since infectious pathogens normally thrive during natural disasters, civil unrest or economic upheaval [26]–[28]. The relationship between infection and atopic diseases has been subject of many studies and originates from the so-called hygiene hypothesis which could explain our results. According to this hypothesis, early childhood infections can down-regulate inflammatory immune responses, thereby suppressing allergic disorders [29]. The rise in infectious disease rates during the 1990 s may thus as such have had an attenuating effect on the development of atopic diseases as observed in our study.

The other major health trend during Cuba’s special period was the declining nutritional status of the population with caloric restrictions, marginal vitamin deficiencies in children and high anemia rates in infants and pregnant women [3], [5]. Several studies have been carried out and different hypotheses have been put forward on the relationship between nutritional status and atopic diseases. According to the Barker hypothesis, undernutrition in early-life, by altering the body’s metabolism, is positively associated with (risk factors for) chronic diseases in adulthood in general [10], [30], [31] and with asthma specifically by impairing lung development [32], [33]. Furthermore, several dietary hypotheses postulate that diet changes, e.g. reduced antioxidant intake, increases the risk for asthma and other atopic diseases, but the available evidence is inconclusive [34], [35]. Neither of these hypotheses are in line with our study results, possibly due to differences in study groups, i.e. schoolchildren from a resource poor country versus adults and populations from resource rich countries, respectively. A number of studies have been devoted to the relationship between obesity and the occurrence of asthma, suggesting that obesity increases the risk of asthma, although the underlying mechanisms are still unresolved [36], [37]. In Cuba obesity decreased during the 1990 s [6] and thus may have been accompanied by a decrease in asthma, but this does not necessarily explain our findings that children that were possibly exposed to a period of undernutrition had lower odds of developing atopic diseases than those unexposed.

The hygiene hypothesis and the relationship between nutrition and atopic diseases are usually considered separately. However, infection and undernutrition are closely related and share a similar geographical distribution, with the same individuals often experiencing both disease states simultaneously [38]. Their co-existence has been explained by two causal pathways: infection leads to undernutrition and alternatively undernutrition increases susceptibility to infection [38], [39], with a strong involvement of the immune system [38], [40], which in turn underlies atopic disease pathology [41], [42]. The observed inverse relationship between atopic diseases and exposure to the economic situation in the nineties of our study group may thus well be the result of some immuno-regulated effect of a synergistic interplay between infection and undernutrition on the development of atopic disease. To our knowledge no studies have been carried out so far about the effect of concurrent undernutrition and infection on atopic disease.

Within the limitations of an ecological analysis, our findings indicate an inverse relationship between exposure to the Cuban economic situation in the nineties during infancy and early childhood and asthma and allergic rhinoconjunctivitis occurrence later in childhood. These results suggest that factors related to this period may have an attenuating effect on atopic disease development. We hypothesized that increased levels of infectious disease incidence and undernutrition during this special period may have been influential factors, either separately or concurrently. However, the exact cause and underlying mechanisms for the observed relationship need to be further elucidated.

## Supporting Information

Table S1 **Adjusted odds ratio’s (OR) with 95% confidence intervals (CI) of exposure to the Cuban economic situation in the nineties for the different atopic diseases if cut-off date is shifted three or six months forward.**
(DOC)

Table S2 **Adjusted odds ratio’s (OR) with 95% confidence intervals (CI) of exposure to the Cuban economic situation in the nineties for the different atopic diseases if transition groups around the cut-off dates are removed.**
(DOC)
