# Supplementary material for: Childhood Atopic Diseases and Early Life Circumstances: An Ecological Study in Cuba
Source: PLoS One. 2012 Jun 29;7(6):e39892. doi: 10.1371/journal.pone.0039892 (PMC3387214; doi:10.1371/journal.pone.0039892)
Supplement: Table S1 — Adjusted odds ratio’s (OR) with 95% confidence intervals (CI) of exposure to the Cuban economic situation in the nineties for the different atopic diseases if cut-off date is shifted three or six months forward. (DOC) [file pone.0039892.s001.doc]

#### Table S1. Adjusted odds ratio’s (OR) with 95% confidence intervals (CI) of exposure to the Cuban economic situation in the nineties for the different atopic diseases if cut-off date is shifted three or six months forward.

|  | | **3 months later** |  | **6 months later** |  |
| --- | --- | --- | --- | --- | --- |
|  | | **Adjusted OR (95% CI)^*^** | ***P*-value** | **Adjusted OR (95% CI)^*^** | ***P*-value** |
| Asthma | |  |  |  |  |
|  | *Unexposed* | 1.0 |  | 1.0 |  |
|  | *Exposed during infancy* | 0.64 (0.37-1.09) | *0.10* | 0.88 (0.53-1.48) | 0.63 |
|  | *Exposed during infancy and early childhood* | 0.49 (0.20-1.22) | 0.12 | 0.76 (0.31-1.83) | 0.53 |
| Allergic rhinoconjunctivitis | |  |  |  |  |
|  | *Unexposed* | 1.0 |  | 1.0 |  |
|  | *Exposed during infancy* | 0.55 (0.30-1.02) | *0.06* | 0.55 (0.30-1.01) | *0.06* |
|  | *Exposed during infancy and early childhood* | 0.37 (0.13-1.06) | *0.06* | 0.35 (0.12-0.99) | **0.047** |
| Atopic dermatitis | |  |  |  |  |
|  | *Unexposed* | 1.0 |  | 1.0 |  |
|  | *Exposed during infancy* | 0.99 (0.45-2.14) | 0.97 | 0.97 (0.46-2.06) | 0.93 |
|  | *Exposed during infancy and early childhood* | 0.81 (0.22-3.03) | 0.76 | 1.02 (0.28-3.67) | 0.98 |

Statistically significant associations are given in bold and borderline significant associations in italic.

* Adjusted for age & municipality.
