# Supplementary material for: Childhood Atopic Diseases and Early Life Circumstances: An Ecological Study in Cuba
Source: PLoS One. 2012 Jun 29;7(6):e39892. doi: 10.1371/journal.pone.0039892 (PMC3387214; doi:10.1371/journal.pone.0039892)
Supplement: Table S2 — Adjusted odds ratio’s (OR) with 95% confidence intervals (CI) of exposure to the Cuban economic situation in the nineties for the different atopic diseases if transition groups around the cut-off dates are removed. (DOC) [file pone.0039892.s002.doc]

**Table S2.** Adjusted odds ratio’s (OR) with 95% confidence intervals (CI) of exposure to the Cuban economic situation in the nineties for the different atopic diseases if transition groups around the cut-off dates are removed.

|  | | **6 months transition** |  |
| --- | --- | --- | --- |
|  | | **Adjusted OR (95% CI)^*^** | ***P*-value** |
| Asthma | |  |  |
|  | *Unexposed* | 1.0 |  |
|  | *Exposed during infancy* | 0.61 (0.27-1.34) | 0.22 |
|  | *Exposed during infancy and early childhood* | 0.64 (0.18-2.31) | 0.49 |
| Allergic rhinoconjunctivitis | |  |  |
|  | *Unexposed* | 1.0 |  |
|  | *Exposed during infancy* | 0.36 (0.14-0.89) | **0.03** |
|  | *Exposed during infancy and early childhood* | 0.26 (0.06-1.17) | *0.08* |
| Atopic dermatitis | |  |  |
|  | *Unexposed* | 1.0 |  |
|  | *Exposed during infancy* | 0.50 (0.15-1.62) | 0.25 |
|  | *Exposed during infancy and early childhood* | 0.48 (0.07-3.21) | 0.45 |

Statistically significant associations are given in bold and borderline significant associations in italic.

* Adjusted for age & municipality.
